# What to do when there is nothing to do? Toward a better understanding of idle time at work

**DOI:** 10.1007/s12144-021-02445-5

**Published:** 2021-11-12

**Authors:** Karoline Schubert, Martin Zeschke, Hannes Zacher

**Affiliations:** Institute of Psychology – Wilhelm Wundt, Leipzig University, Neumarkt 9-19, 04109 Leipzig, Germany

**Keywords:** Idle time, Proactive behavior, Adaptive behavior, Work constraints, Action regulation theory

## Abstract

Idle time at work is a phase of involuntary downtime during which employees experience that they cannot carry out their work tasks. In contrast to breaks, interruptions, procrastination, or withdrawal behavior, employees cannot work because of the absence of work-related tasks. Based on action regulation theory, we develop an integrative conceptual model on the antecedents and consequences of the subjective experience of idle time. We propose that work constraints (i.e., regulation problems) have negative effects on occupational well-being and task performance, and that these effects are mediated by subjective idle time. The strength of these effects is further assumed to be influenced by individuals’ use of proactive (i.e., prevention) and adaptive (i.e., coping) strategies. Results of a supplemental qualitative study, for which we interviewed 20 employees from different occupations, provided preliminary support for the propositions. Finally, we develop theory on how individual, situational, and organizational characteristics may influence the proposed effects on and of idle time. Overall, this conceptual development paper contributes to a better theoretical understanding of idle time at work by extending its definition and applying action regulation theory to this practically important phenomenon.

## Introduction

The question of whether 40 h of work per week, in the context of paid employment, is “too much” is one that has arisen not just recently (Greene, 2018). The 40-h work week is considered a tradition in many countries (Lee et al., 2007) and, even though work has changed significantly as a result of globalization, digitalization, and flexibilization, this does not seem to have had an impact on average working hours (Lehndorff & Hermann, 2017). The fact that working hours were not reduced over the last few decades could mean one of two things: Either the saved time is now filled with newly created work tasks or the free time remains idle. A European survey of 10,000 employees suggested that 33% had not enough work to do and 30% never worked toward tight deadlines (Green & McIntosh, 2001; Rothlin & Werder, 2007). Consistently, a survey among German white-collar workers found that 57% reported a preference for a reduction in working hours instead of a salary increase (Stuth, 2019). Indeed, companies that have reduced working hours (while maintaining the salary) reported increased productivity (e.g., Pang, 2020). Swedish scholars found similar results. One study among social workers and one among nurses showed that reduced working hours led to improved well-being, sleep quality, and less stress (Åkerstedt et al., 2001; Barck-Holst et al., 2017).

These findings suggest that somewhere in a “9 to 5” workday, in many cases there is time that is not filled with work. Prior research on non-work periods at work has generally focused on two strands: On the one hand, there are work breaks. These are voluntary non-work phases at work that can be considered on-the-job recovery experiences (Hunter & Wu, 2016; Kühnel et al., 2016). Breaks have been found to have positive effects on well-being and performance (Fritz et al., 2013; Fritz et al., 2011). On the other hand, there are interruptions at work. Interruptions are caused by external factors residing at different levels (e.g., organizational rules and procedures, job characteristics, and task related reasons, such as technical malfunctions; Paulsen, 2014; Solebello, 2016). Research has shown that interruptions have negative effects on well-being and performance (Baethge et al., 2014).

However, somewhere in between these two strands, there is another form of non-work at work that research has neglected: idle time. So far, hardly any conceptual or empirical research focuses on this phenomenon, with three exceptions: First, Brodsky and Amabile (2018) examined the prevalence of idle time and some of its potential consequences (e.g., performance). They defined idle time as “involuntary downtime during which in-role tasks cannot be done” (p. 3). Based on a representative online survey, they found that most U.S. employees across different occupations experienced idle time at least once a month. In addition, they estimated that 21.7% of employees experienced idle time daily. Anecdotal evidence suggests that even some employees in highly-paid jobs have too little to do (Pennekamp & Jung, 2020). Moreover, idle time seems to have major financial consequences for employers. Brodsky and Amabile (2018) estimated 7.4 billion “empty” work hours per year, causing a 100-billion-dollar financial loss. Second, in another recent study on idle time, Lei et al. (2019) investigated potential individual consequences of idle time and found that it is a negative subjective experience common among employees, and that it can have negative consequences for job satisfaction, job performance, and subjective health. Third, Phillips (2016) conducted an observational study in a police agency to explore the behaviors that police officers engage in to decrease their feelings of boredom during idle time. Results showed that officers typically pursued activities during idle time that were expected of them (e.g., patrolling troubled neighborhoods, backing up other officers). However, despite these few studies, idle time remains conceptually and empirically a neglected phenomenon in the work and organizational psychology literature. In particular, what is still missing is a theoretical framework of the nomological network of idle time at work. We need to clarify the nature of idle time, understand why and when it occurs, and how employees can prevent or cope with it.

In this paper, we address these issues by providing a comprehensive definition of idle time and by introducing the duality of objective and subjective idle time. Furthermore, we argue that idle time can be conceptually differentiated not only from breaks and interruptions, but also from deliberate non-working behaviors at work, such as procrastination and withdrawal behavior. We criticize that research so far has approached the concept of idle time mainly empirically and practically. Therefore, we develop an integrative conceptual model on idle time based on action regulation theory, a meta-theory of the psychological regulation of goal-directed behaviors at work (Frese & Zapf, 1994; Zacher & Frese, 2018). To provide initial support for our theoretical considerations, we report the results of a qualitative study for which we conducted 20 semi-structured interviews with employees from different occupations.

The contribution of this conceptual development paper is threefold. First, we show that previous research on non-work periods at work has neglected the theoretically and practically important phenomenon of idle time. We establish idle time as a unique construct that is independent of breaks and interruptions and suggest the duality of objective and subjective idle time. Second, we lay the foundation for further empirical research on idle time by developing an integrative conceptual model that addresses antecedents, consequences, and behavioral strategies that may help to prevent or cope with idle time. Additionally, we provide suggestions on how individual, situational, and organizational characteristics might influence idle time and how future studies could address these influences. Third, we appeal to future work design efforts that aim to reduce the prevalence and consequences of idle time for individuals and organizations. Designing work in a way that helps individuals to prevent idle time or use it meaningfully could reduce its negative consequences for both employees and companies. Thus, based on the theoretical foundation laid out in this paper, employment conditions could be implemented that involve that people are paid for their performance at work instead of being paid for staying at work for the fixed amount of time of 40 hours a week.

## The Nature of Idle Time at Work

### Definition

Idle time has been defined as “involuntary downtime during which in-role tasks cannot be done” (Brodsky & Amabile, 2018, p. 3). A key aspect of this definition is that idle time has to be involuntary, in that it occurs due to external factors, while employees are generally capable and willing to work (Lei et al., 2019). Thus, employees have the ability and motivation to work, but they have no opportunity to do so, which has been described as a key prerequisite of job performance (Blumberg & Pringle, 1982). Idle time is a work situation without any available tasks to be completed. Thus, task performance is blocked. Idle time periods have been characterized by the absence of work tasks (“no work is available,” Brodsky & Amabile, 2018, p. 3; “lack of any pressing taskwork,” Lei et al., 2019, p. 361) and represent the opposite of periods of high workload (“opposite of intense busyness,” Brodsky & Amabile, 2018, p. 3; “low workload periods,” Lei et al., 2019, p. 361).

Idle time is likely to occur when work constraints are present, however, these constructs have to be conceptually differentiated. Work constraints are *situational characteristics* that hinder employees from successfully accomplishing their work tasks, including the lack of information or feedback, missing customers, or technical breakdowns (Peters & O'Connor, 1980; Pindek et al., 2019). The occurrence of work constraints is followed by a *work situation* where employees are not able to carry out their work tasks due to reasons beyond their control. Thus, work constraints are *working conditions*, whereas idle time is a *situation at work* characterized by the absence of work tasks. Idle time, therefore, is an immediate consequence of work constraints. Even if idle time always follows from work constraints, it does not mean that they are the same – they are temporally separated, in that idle time occurs after work constraints. However, idle time does not necessarily have to be recognized as such, as employees may not think that they have nothing to do. Therefore, we propose a duality of idle time*. Objective idle time* refers to the situation when employees are unable to fulfill their core job tasks. In contrast, *subjective idle time* is employees’ psychological experience that they, in the present moment or for a certain time period, are unable to work on their core job tasks. Subjective idle time thus represents employees’ judgment that there is no possibility of completing in-role tasks during a certain period of time. For example, imagine a salesman without customers. The lack of customers represents a work constraint that leads to a situation where no work tasks are available (objective idle time). If he realizes that there is nothing to do, he will experience subjective idle time. Another example might be a researcher whose computer broke down, and this work constraint hinders her to finish a paper. We decided to focus on subjective idle time in our conceptual model because objective idle time is always a direct consequence of work constraints. Therefore, we do not refer to objective idle time in the conceptual model developed below. We address work constraints as ultimate determinants and explain how people then experience the following situations as idle.

### Differentiation from Similar Constructs

Two aspects of its definition help to distinguish idle time from similar concepts: idle time is a work situation that has to be involuntary, and during which it is impossible to fulfill work tasks. This differentiates idle time from other work situations or behaviors at work in which tasks are not completed because employees have to or want to focus on another task or non-work activity for some time. These situations and behaviors, including breaks, interruptions, procrastination, and withdrawal behavior have been the focus of previous investigations of non-working periods at work (e.g., Baethge et al., 2014; Fritz et al., 2013; Paulsen, 2014; Steel, 2007). Table 1 summarizes definitions of each of these constructs and provides an overview of dimensions that help to differentiate idle time.

**Table 1:** Definitions and dimensions of idle time and related constructs

		Dimensions		
	Definition	Initiation	Availability of work tasks	Manifestation
Idle time	“A period of involuntary downtime during which in-role tasks cannot be done” (Brodsky & Amabile, 2018, p. 3)	external/involuntary	no work tasks available	time-period
Breaks	“Periods of downtime during which work is available but is not expected to be done” (Brodsky & Amabile, 2018, p. 3)	internal/voluntary	work tasks available	time-period
Interruptions	“An interruption is a temporary suspension of a person’s goal-directed action” (Baethge et al., 2014, p. 2)	external/involuntary	work tasks available	time-period
Procrastination	“We procrastinate when we delay beginning or completing an intended course of action” (Steel, 2007, p. 6)	internal/voluntary	work tasks available	behavior
Withdrawal behavior	“Employees’ avoidance of or disengagement from the work environment, tasks, or the organization” (Carpenter & Berry, 2017, p. 835)	internal/voluntary	work tasks available	behavior

First, idle time occurs due to *external factors*. Its onset is beyond employees’ personal control. Breaks, in contrast, are mostly initiated by internal factors and are started voluntarily (Jett & George, 2003). For work breaks, the initiators can be the employees themselves or their organization, which may schedule formal break times. This does not necessarily mean, however, that recovery-related experiences and behaviors (e.g., relaxation) are not applicable during idle time. Similar to breaks, during procrastination and withdrawal behavior, the initiators of downtime are the employees themselves (Paulsen, 2014; Steel, 2007). Employees voluntarily decide not to work. Both are initiated internally and are, therefore, voluntary behaviors.

Second, independent of people’s own motivation, skills, and abilities to work, during idle time *no work tasks are available*. During breaks, interruptions, procrastination, and withdrawal behavior, individuals decide to or are asked to focus on another task or non-work activity for a certain period of time (Baethge et al., 2014; Fritz et al., 2013; Paulsen, 2014; Steel, 2007). Additionally, procrastination and withdrawal are not situations, but employee behaviors. Breaks, interruptions, and idle time are conceptualized as *time periods during work* (Jett & George, 2003). Procrastination and withdrawal behavior, in contrast, are behaviors that people engage in while at work.

In summary, idle time is a period at work during which work-related tasks cannot be carried out due to reasons beyond personal control, which differentiates idle time from similar constructs, including breaks, interruptions, procrastination, and withdrawal behavior.

## Conceptual Model and Propositions on Idle Time at Work

**Fig. 1:**
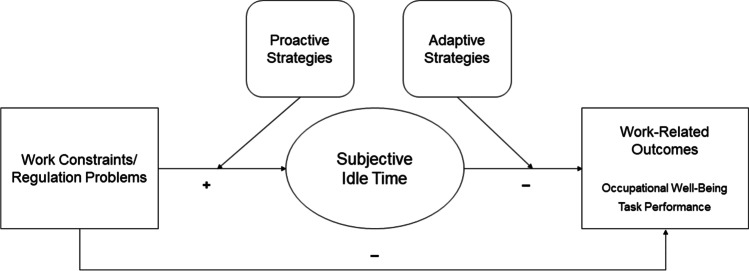
Conceptual model of idle time at work

In this section, we develop an integrative conceptual model on the antecedents and consequences of idle time. With this model, we address three overarching questions: (a) Which factors increase the likelihood of idle time at work? (b) What is the impact of idle time at work on important employee outcomes? (c) How can these processes be influenced by employees? The conceptual model and propositions are graphically summarized in Fig. 1. In the following, we develop and justify the propositions in further detail, drawing from a well-established theoretical framework, action regulation theory (Frese & Zapf, 1994; Hacker, 1998).

Action regulation theory is a meta-theoretical framework that is concerned with the initiation and pursuit of goal-directed behavior in the work context. Humans are conceived as active agents who tend to influence their dynamically changing environment to achieve their goals, that is, their internal representations of desired states (Austin & Vancouver, 1996). Action regulation theory explains the psychological regulation of behavior through sequential cognitive processes, including goal development and selection, planning and monitoring of actions, and processing feedback (Zacher, 2017; Zacher & Frese, 2018). Action regulation occurs at different hierarchically organized levels: the sensorimotor or skill level, the level of flexible action patterns, the intellectual level, and the meta-cognitive heuristic level. The long-term cognitive representation of the relations between actions, boundary conditions, and action results is the action-oriented mental model (Zacher & Frese, 2018). These representations include unconscious movement-oriented schemata; flexible, but routinized action patterns; conscious, complex, and intellectual schemata and strategies; and meta-plans and heuristics (Frese & Zapf, 1994). Action-oriented mental models help employees through the process of action regulation until goals are achieved, and are fundamental for efficient and effective action regulation (Frese & Zapf, 1994).

Employees work most efficiently when work tasks are sequentially and hierarchically complete. Sequentially complete tasks enable individuals to experience all phases of action regulation and hierarchically complete tasks enable them to regulate their behavior on all mental levels. This allows employees to use different skills and abilities to achieve their work goals, which helps to improve their competencies and increase work motivation and performance (Zacher & Frese, 2018). Thus, according to action regulation theory, work design should provide sufficiently complex tasks (i.e., regulation requirements) and control (i.e., regulation possibilities) to enable individuals to actively cope with their environment (Zapf, 2002). Work stressors and constraints (i.e., regulation problems), on the other hand, should be reduced (Zapf, 2002).

### Work Constraints and Idle Time

Idle time occurs when people are unable to fulfill their in-role tasks due to work constraints that hinder individuals from fulfilling their work tasks. In action regulation theory, work constraints are referred to as regulation problems that disturb the regulation of actions and can be further categorized into regulation obstacles, regulation uncertainty, and overtaxing regulation (Frese & Zapf, 1994).

*Regulation obstacles* are defined as situations and factors that make it difficult or impossible to attain an action goal and, thus, to complete ongoing tasks, including lack of information, poor material, or interruptions by either other persons or technical (e.g., computer breakdown) and organizational problems (e.g., lack of supplies; Frese & Zapf, 1994). *Regulation uncertainty* includes missing information about how to achieve a goal, missing or delayed feedback, as well as role ambiguity or conflict (Frese & Zapf, 1994; Zapf, 1993). The third category of regulation problems refers to *overtaxing regulation*, for example, high levels of time pressure or information overload. The difficulty arising from overtaxing regulation is the speed and intensity of regulation (Frese & Zapf, 1994). Overtaxing regulation does not constitute a prerequisite of idle time because it makes it still possible to achieve work goals, which is per definition impossible during idle time (i.e., work-related tasks cannot be accomplished).

High levels of regulation obstacles and regulation uncertainty can hinder individuals from fulfilling their in-role tasks. They overlap conceptually with the construct of work constraints. Peters and O'Connor (1980) propose different constraints, which impede the use of abilities to fulfill work demands. These constraints include organizational rules and procedures, lack of information, incorrect instructions, and a lack of or poor equipment or supplies. Examples are malfunctions or breakdowns of machines or computers, as well as download or updating time (Brodsky & Amabile, 2018; Peters & O'Connor, 1980). Referring to the examples above, a work constraint of the salesman would be the absence of customers, whereas for the researcher it could be a computer breakdown. For reasons of consistency, we use the term work constraints from now on to refer to both regulation problems and constraints that disturb the action regulation process and make it more difficult or impossible to achieve a goal (i.e., fulfill a work task; Zacher & Frese, 2018). Our first proposition, accordingly, is that work constraints are crucial for idle time at work to occur.

- *Proposition 1:* Higher levels of work constraints are associated with higher levels of subjective idle time at work.

### Idle Time and Employee Outcomes

Work constraints are associated with higher levels of frustration and dissatisfaction and lower performance (Peters & O'Connor, 1980). Their absence results in a more positive mood (Peters & O'Connor, 1980) and higher job satisfaction (Kovner et al., 2006). However, these associations have not been rigorously tested so far (Pindek et al., 2019). Moreover, the role of idle time in this association has not been elaborated.

We assume that a high level of work constraints negatively influences occupational well-being and performance. Occupational well-being entails subjective experiences at work, such as high positive (e.g., vigor, enthusiasm) and low negative (e.g., exhaustion, frustration) affect, as well as subjective feelings of meaning at work, learning, and growth (Sonnentag, 2015). With regard to performance, we apply the multidimensional conceptualization of job performance that entails task performance, citizenship performance, and counterproductive performance as key components (Rotundo & Sackett, 2002). More specifically, we focus on task performance in our conceptual model, which refers to “behaviors that contribute to the production of a good or the provision of a service” (Rotundo & Sackett, 2002, p. 67). Task performance thus refers to the extent to which a worker successfully carries out their core work tasks. In our conceptual model, we include task performance as an outcome variable because the focus of task performance and idle time is the fulfillment of core job tasks (i.e., in-role tasks). In contrast, citizenship and counterproductive performance refer to behaviors that are not task-related (i.e., extra role tasks; Rotundo & Sackett, 2002). Therefore, they are not discussed as outcome variables here because they are considered as workers’ potential behavioral strategies for dealing with idle time at work (see “The Role of Proactive and Adaptive Strategies” below).

According to action regulation theory, work constraints should affect both task performance and occupational well-being, because work constraints impede the action regulation process (Zacher & Frese, 2018). Incomplete or fragmented regulation of action can lead to feelings of frustration and boredom and diminish competencies (Zacher, 2017). In contrast, sequentially and hierarchically complete tasks enable employees to use different skills and abilities on all mental levels to achieve their work goals. Goals motivate employees and increase task performance. Moreover, an incomplete or fragmented action regulation process requires the investment of resources and cognitive effort by employees (Baethge et al., 2014). Diminished resources are associated with lower well-being and performance (Hobfoll, 1989; Zacher, 2017). Evidence supports the direct link between work constraints and occupational well-being (de Lange et al., 2003; Peters et al., 1988) and task performance (Fritz & Sonnentag, 2009; Gilboa et al., 2008).

- *Proposition 2:* Higher levels of work constraints are associated with (a) decreased occupational well-being and (b) decreased task performance.

We expect that the direct links between work constraints and occupational well-being and task performance are mediated by subjective idle time. We focus on subjective idle time because we assume that this psychological experience, and not only an idle work situation per se, is a prerequisite for changes in occupational well-being and task performance. In other words, objective idle time alone (without employees perceiving subjective idle time) should not be sufficient to impact work-related outcomes.

Subjective idle time can be conceptualized as a highly aversive experience that will be avoided by employees if somehow possible (Brodsky & Amabile, 2018; Weiss & Cropanzano, 1996). The personal assessment that there is no possibility to complete in-role tasks and achieve work goals during a certain period of time undermines the natural human tendency to act (Zacher & Frese, 2018). In a qualitative study, Lei et al. (2019) explore the unpleasantness of idle time. The results of interviews showed that idle time is a phase during which employees felt bored and tired, and that they preferred busy times at work. Moreover, in an experimental study, Wilson et al. (2014) found waiting time, a construct similar to idle time, to be so aversive that participants even gave themselves electric shocks to avoid being left alone with nothing to do. Another study found relationships between waiting time and negative affect (Antonides et al., 2002). Thus, subjective idle time is an aversive experience that increases negative affect and adds to the diminishing influence of work constraints on occupational well-being.

- *Proposition 3a:* The negative effect of work constraints on occupational well-being is mediated by the subjective experience of idle time.

Individuals prefer to act instead of doing nothing (Lei et al., 2019). According to action regulation theory, employees work most efficiently when they use different skills and abilities to achieve their work goals (Zacher, 2017; Zacher & Frese, 2018). That way, they experience sequential and hierarchical completeness during the action regulation process. Evidence supports these expected negative effects of subjective idle time on task performance (Kuem & Siponen, 2014), including slower working pace (Brodsky & Amabile, 2018; Dempsey et al., 2010; Latham & Locke, 1975) and poorer in-role work behavior (Latham & Locke, 1975; Lei et al., 2019). In a daily diary study, Lei et al. (2019) found the amount of idle time to be negatively related to job performance. In a set of experimental studies, Brodsky and Amabile (2018) emphasized the effects of idle time on working pace. They manipulated the expectation of time without tasks and the possibility to use the time for personal smartphone use. They found slower working paces in the idle time group compared to the working group. The possibility to use the time for personal internet activities fastened participants working pace (Brodsky & Amabile, 2018). Thus, the subjective experience of idle time without any intervening behavior should affect task performance negatively.

- *Proposition 3b:* The negative effect of work constraints on task performance is mediated by the subjective experience of idle time.

### The Role of Proactive and Adaptive Strategies

Objective idle time is an immediate consequence of work constraints. However, employees will not necessarily experience subjective idle time when they are confronted with work constraints. Employees could attempt to actively prevent the subjective experience of idle time. Using the example of the researcher, she could write a to-do list for her next projects or ask co-workers for feedback on her ideas during the computer breakdown instead of perceiving idle time. If it is not possible for her to engage in such preventive actions, she could try to deal with the experienced idle time by relaxing or using her smartphone for private activities. Thus, employees who cannot prevent idle time could cope with it. Preventive actions are proactive behaviors (Parker et al., 2006), whereas coping actions are adaptive behaviors (Brown et al., 2005).

#### Proactive Strategies

Brodsky and Amabile (2018) suggested that expected or anticipated idle time evokes different reactions or work behaviors than unexpected idle time. The anticipation of imminent idle time helps individuals to prevent its subjective experience. Action regulation theory states that individuals actively regulate their actions to adapt to (anticipated) changes in the work environment (Kooij et al., 2020; Zacher & Frese, 2018). Work constraints and the following idle time represent a salient change in the work environment. Whereas control theory suggests that individuals tend to react to or cope with stress arising from work constraints, individuals also proactively deal with stressors and prevent long-term consequences (Edwards, 1998; Fay & Sonnentag, 2002). Employees should be generally motivated to regulate and influence their work environment, and avoid the aversive experience of idle time by engaging in any activity at work even outside the scope of in-role tasks (Lei et al., 2019; Zacher & Frese, 2018). Proactive behavior constitutes the anticipation and prevention of problems by engaging in active, future-, and change-oriented actions (Parker et al., 2010). Work constraints are positively associated with proactive behavior (Fay & Sonnentag, 2002; Fritz & Sonnentag, 2009) because they motivate employees to reveal what could be improved in the workplace (Sonnentag & Spychala, 2012). Additionally, longitudinal data showed that proactive behavior affects decreases in work constraints (Li et al., 2014). Even if work constraints could not be removed, individuals seek more favorable work environments (A. M. Grant & Parker, 2009). The use of proactive behavioral strategies can prevent the experience of idle time and, thus, possible negative effects on occupational well-being and task performance could be diminished.

Two exemplary proactive strategies are job crafting strategies and work stretching. A construct very similar to job crafting is the notion of citizenship performance, which entails contributing to the achievement of the organization’s goals by influencing the social and psychological work environment (Rotundo & Sackett, 2002). Job crafting also refers to different aspects of the job, including task-related, social, or cognitive aspects (Rudolph et al., 2017; Wrzesniewski & Dutton, 2001). First, task-related aspects could include learning something new, setting new goals, or making a to-do list (Fritz et al., 2011). Fritz et al. (2011) found these work-related strategies to have positive relations with vitality. Additionally, Fisher (1993) described goal setting to have a decreasing effect on boredom. Second, a change in relational aspects of work could include asking for feedback or helping a coworker (Bakker & Demerouti, 2017; Fritz et al., 2011). Talking to a coworker or supervisor is one of the three most commonly used work-related energy management strategies at work (Zacher et al., 2014). Third, job crafting can involve cognitive changes, such as reflecting on different work aspects or thinking about the meaning of one’s job (Bakker & Demerouti, 2017; Fritz et al., 2011).

Another proactive strategy is work stretching. Work stretching is the tendency to work more slowly in the absence of tight deadlines and is based on pacing theory (Mitchell et al., 2008). This theory states that employees adapt their work pace to the time available to complete their tasks. Working more slowly might not seem proactive at first sight but proactive strategies include the anticipation and active prevention of unfavorable working conditions (Fay & Sonnentag, 2002). The experience of idle time is unfavorable and can be prevented by stretching prior work tasks to keep active and busy and impede aversive idle time (Brodsky & Amabile, 2018; Wilson et al., 2014). Regarding the example of the salesman, he could use work stretching by taking a lot of time to serve a single customer when he expects to experience idle time again after this one. In an experimental design, Brodsky and Amabile (2018) found that participants expecting idle time slowed down their work pace as the task progressed on a greater level than participants without the expectation of idle time. Thus, if idle time could be anticipated, participants were more likely to work slower to avoid unpleasant downtime (Brodsky & Amabile, 2018).

In summary, we assume that the natural human tendency to actively influence the environment would, in many cases, lead to the use of proactive behavioral strategies that prevent employees from experiencing idle time. The positive association between constraints and proactive behavior supports this suggestion. We therefore expect that the extent to which employees use proactive behavioral strategies moderates the impact of work constraints on subjective idle time, such that the use of proactive strategies makes subjective idle time less likely.

- *Proposition 4:* The use of proactive behavioral strategies moderates the positive relationship between work constraints and subjective idle time, such that the relationship is weaker when employees use proactive strategies and stronger when they do not use proactive strategies.

#### Adaptive Strategies

If proactive behavioral strategies are not applied or fail to prevent idle time, employees could make use of adaptive strategies. In contrast to proactive strategies, which include behaviors that actively shape the environment, adaptive strategies are used to react to changes in the environment (Fay & Sonnentag, 2002; Kooij et al., 2020). This means that employees do not influence their work environment, but instead adapt their behavior to the situation to cope with it. As noted by Folkman and Lazarus (1988, p. 466), “any one stressful event [...] usually has more than one implication for well-being and more than one option for coping.” In the current context, adaptive behavioral strategies involve any behavior of an employee that leads to an adaption to the subjective experience of idle time. Drawing from action regulation theory, employees could adapt their goals by no longer trying to fulfill a work task but, instead, by trying to cope with idle time (Zacher & Frese, 2018). Adaptive coping strategies help to react to and deal with stress, and thus, buffer negative effects on well-being (Edwards, 1998; Fay & Sonnentag, 2002). However, this buffer effect does not apply to task performance because there are no work tasks present during idle time. Coping seeks to react to unpleasant changes in the environment (Edwards, 1998). It is not directed towards work-related activity but towards restoring resources. Thus, we assume that the use of adaptive strategies moderates the relationship between subjective idle time and occupational well-being.

Two exemplary adaptive behaviors are recovery strategies and cyberloafing (e.g., Mercado et al., 2017; Sonnentag et al., 2017). Recovery refers to the process of counteracting demanding aspects of work (e.g., idle time) and consequently strain (Sonnentag et al., 2017). Generally, studies show that break periods at work positively affect well-being (see Fritz et al., 2013 for an overview). Recent research also examined informal breaks during work time to have positive effects on well-being (Hunter & Wu, 2016; Rhee & Kim, 2016; Trougakos et al., 2008). Especially the experience of psychological detachment and relaxation decreases psychological strain (Sonnentag et al., 2017). Zacher et al. (2014) examined the role of micro-breaks on occupational well-being and found a positive relationship with vitality and a negative relationship with fatigue. Micro-breaks refer to activities such as listening to music, daydreaming, or making plans for the evening (Fritz et al., 2011). One recent daily diary study investigated the role of different types of activities moderating the negative effects of idle time on occupational well-being (Lei et al., 2019). They found recovery activities to buffer the negative effect of idle time on work fatigue.

Because most employees have the opportunity to use information technology devices, cyberloafing is another common activity during idle time (Mercado et al., 2017). Cyberloafing is an employee’s non-work behavior that involves using information and communication technologies instead of completing work tasks (Mercado et al., 2017). Cyberloafing does not apply for all kind of jobs but affects various organizations. As the term “loaf” (to avoid activity, especially work) suggests, most studies focused on cyberloafing as a form of counterproductive work behavior (e.g., Block, 2001; Smale, 2016). Indeed, counterproductive work behavior is thought to be a consequence of constraints (Spector & Fox, 2010) or a component of the job performance domain, with harmful consequences for other people and organizations (Rotundo & Sackett, 2002). In our model, however, we focus on those strategies that help mitigate negative effects of idle time on occupational well-being. Thus, cyberloafing can be counterproductive for employees who have work to do, but not necessarily for those who experience idle time (Mercado et al., 2017). Cyberloafing has been suggested to serve as a coping or recovery strategy during idle time (Pindek et al., 2019). To avoid boredom at work caused by work underload, employees are much more likely to engage in cyberloafing as a coping behavior than in other (counterproductive) work behaviors (Pindek et al., 2019).

In summary, we assume that individuals engage in adaptive strategies if the experience of idle time could not have been proactively prevented. Employees would adapt their own behavior and respond to the working environment by changing their goals. They would shift their attention from fulfilling work tasks to dealing with idle time. We expect the use of these behavioral strategies to moderate the impact of subjective idle time on occupational well-being, not on task performance, because adaptive strategies are aimed at rebuilding resources rather than facilitating work processes. They are applied to cope with idle time, not to prevent it or fill it with work-related actions.

- *Proposition 5:* The use of adaptive behavioral strategies moderates the negative relationship between subjective idle time and well-being, such that the relationship is weaker when adaptive strategies are used and stronger when adaptive strategies are not used.

## Results of a Qualitative Study

We conducted a supplemental qualitative study to gain a deeper understanding of the idle time construct and to provide preliminary support for our conceptual model and associated propositions. We designed a semi-structured interview manual based on the conceptual model with questions aimed at exploring our central constructs and propositions. The interview manual, the coding schemes, the interview transcripts, and questionnaire data can be found on the Open Science Framework https://osf.io/9xm7t/.

### Data Collection and Analysis

For recruitment, we employed a convenience sampling approach. To this end, we shared the research topic of idle time with people in our personal and professional networks and, in this way, recruited participants who could identify with such work situations. Convenience sampling was deemed a suitable method of recruitment for our purposes, as it ensured that the interviewees were willing to give us deep insights into the reality of their personal and work lives, while trusting us with this sensitive topic. This was especially important because most of the interviews were conducted over the phone (data collection took place during the COVID-19 pandemic, February to April 2020). We ensured that the interviewees were not familiar with our model and propositions beforehand. We informed them in advance that we aimed to conduct interviews on their current work situations with a focus on idle time. We recruited 20 employees (13 female, 7 male) between 20 and 49 years old (*M* = 32.00, *SD* = 9.16). They worked mainly in service industries (see Table 2 for details), and for approximately 35 h a week on average (*M* = 34.73, *SD* = 19.39).

**Table 2:** List of job descriptions/industries sorted by interviewee

Interviewee code	Job description/industries
DS01	Retail trade
DS02	Technician
DS03	Business consulting
DS04	Hair salon management
DS05	Pre-sales management
DS06	Adult education (self-employed)
DS07	Catering trade (self-employed)
DS08	Car production machine maintenance
DS09	Choral singer
DS10	Food production and marketing
LS01	Customer service
LS02	Law enforcement
LS03	Church administration
LS04	Auditing, tax and management consultancy
LS05	Transportation engineering
LS06	Research
LS07	Catering
LS08	Entertainment industry
LS09	Research
LS10	Automotive mechatronics

Data collection consisted of two parts. We started with semi-structured interviews and concluded with a short questionnaire on demographics. Interviews were conducted by two student interviewers, were recorded via phone or voice recorder and were transcribed subsequently with the software MAXQDA 2020 (VERBI Software, 2019). Questionnaires were either answered in paper and pencil format or via an online survey. For the analysis of the interviews we adopted the procedure of the structuring content analysis (Mayring & Brunner, 2006). Specifically, the interview transcripts were coded in MAXQDA using categories for each construct in our conceptual model. Each text passage that included a reference to a construct was labeled with the corresponding category. The first interview (DS01) was coded collaboratively by the two interviewers to establish a shared understanding of the categories. Thereafter, each interviewer coded the interviews they had conducted. Proactive and adaptive strategies in each interview were coded independently by both interviewers and categorized as either proactive or adaptive according to a coding scheme prepared in advance by four subjective matter experts (i.e., graduate students and academics with a work and organizational psychology background). After assigning all categories based on the conceptual model, the interviewers categorized specific text passages into thematically relevant clusters. For instance, a specific cluster of adaptive strategies was cleaning/tidying up the workspace. All interviews were conducted and transcribed in German and we only translated the quotes for this manuscript. In the following, we share the most profound example quotes from a selected group of employees. For additional quotes, please see Table 3.

**Table 3:** Results of the qualitative interviews sorted by construct with corresponding category

Variable	Sample Quotations	Category
Work constraints	DS02: I realize, to get this job done, certain parts are missing.	Lack of material
	DS03: I have to wait for customer information.	Lack of information
	DS04: Nobody booked an appointment.	No customers
	DS05: I cannot continue because certain parts are missing.	Lack of material
	DS07: No customers.	No customers
	LS02: The worst scenario is a system breakdown.	Technical malfunction
	LS05: Most of the time, it’s because of a lack of projects.	Lack of information
Idle time dimensions	DS02: In this specific situation I had to wait two and a half days before the missing parts arrived.	Duration
	DS03: During an 8-to-9-h workday, sometimes 7 h are idle.	Duration
	DS08: 1 to 2 h.	Duration
	DS09: The longest idle time is about 1,5 h.	Duration
Outcomes	DS01: It’s an aversive time, it is like sleeping. It is really … really bad.	Well-being
	DS05: On one hand, I feel bad about getting paid for nothing. On the other hand, I feel useless, needless and bored	Well-being
	DS10: I’m stressed out that the time is not passing by. I am exhausted and sad.	Well-being
	LS08: I’m bored, exhausted, and depressed. It really feels like a depression. I don’t want to go at work in the first place, and when I’m there, I count the minutes until I can finally leave. My whole life quality suffers.	Well-being
	DS03: I really feel ashamed when my project manager is asking me about my progress, and I have to tell him that my hands are tied, and I am incapable of acting. Moreover, I am bored, insecure about being dismissed, unchallenged and I feel unseen, not appreciated.	Well-being, Task performance
	DS04: I feel unchallenged, not working to capacity. I am missing motivation, and, in the evening, I feel more exhausted than on a busy day.	Well-being, Task performance
	DS07: I worry about the future, have fundamental existential fears, and I feel depressed.	Well-being, Task performance
	DS08: I think, I would like to have another job. I just feel … I feel tired. Imagine, you just feel exhausted and sit on a couch, that’s how I feel every day.	Well-being, Task performance
	LS10: It is annoying, I’d rather work for my money.	Task performance
Proactive strategies	DS03: Perhaps I would also postpone fewer tasks and do more immediately, you know? One strategy that I talked about earlier was to save up work for idle times […].	Work stretching
	DS10: I would make phone calls throughout the day and then enjoy the breaks in between. You know? So that I don’t work in a hurry and that I’m finished quickly but rather do it more according to desire and mood I would stretch out the tasks a bit.	Work stretching
	LS01: Previously, I tried to stretch out my work a bit, but then I always had to slow myself down. I said to myself ‘slow down, take your time’.	Work stretching
	LS10: [If] you really have nothing left to do, you automatically work slower. Exactly, artificially, you artificially extend your work.	Work stretching
	DS01: I contact my supervisor and ask for something to do.	Job crafting
	DS03: I would for example write a documentation or talk to my colleagues. They sometimes tell me that I can still do something, for example, if I have time, I might change or program something on the website for the company. Or I asked colleagues to check if they had any work for me, if there was something on the agenda.	Job crafting
	DS04: I clean up work materials from the previous day. Uh, paint trays or something like that. Uh... yeah, then I also sort paint tubes. I just look around for tasks. Otherwise, as I said, I also read specific journals to learn something new […] We want to start writing promotions on it [displayer]. For example ‘New customers get so and so much percent discount’ … things like that, or promotion days.	Job crafting
	DS05: Well, looking for a new job. Switch to another company. I then took care of it myself, so that I, uh, supported other offices that still had properties that could be sold. […] I also did things like organizing everything where I otherwise wouldn’t have had time to do so.	Job crafting
	DS07: I also think about my business, about the work itself. I mean, I think about, what I could do differently. [If] there are no customers, I think about what would makes the most sense to do.	Job crafting
	DS08: We try to educate ourselves internally as much as possible […] We just look for something to do, for example maintaining any lists of material or Excel sheets.	Job crafting
	DS09: I try to recap songs for another play that I had to learn really quickly.	Job crafting
	DS10: Well what do I do during idle time? Uh, I work off my items from the to-do list or I write down certain things. I take a look to the left and to the right. Let’s see, what else can I do […] I think about selling strategies or I read journals […] I always have a phone list with me, things that I need to clarify and otherwise can’t manage. And... yeah, and then there are always a few notes that have to be updated. Or I clean up my boxes a little bit, which I don’t get to do otherwise.	Job crafting
	LS01: [I checked] if I could help my colleagues or if there was the opportunity to talk or chat about work with them. […] Watering flowers, cleaning the office, I don’t know, anything. I try to do anything to not just sit there and wait for the workday to end.	Job crafting
	LS02: Organizing folders and files in a more structured way, or deleting old things. So, it’s not just cleaning out lockers, but also cleaning out directories, that is a very big issue.	Job crafting
	LS10: I’ve been wanting to fix, clean up or sort things here in the factory for a long time […] First, I talk to the supervisor: What else do you have to work on? Do you know anything else to do?	Job crafting
Adaptive strategies	DS03: Whether it’s doing laundry, going to the city center, shopping, or leaving work early […] I go to the kitchen, and get a tea or coffee.	Recovery
	DS04: Then I simply serve my time.	Recovery
	DS08: Well, if there’s still time, we just have a coffee together, chat a bit about our free time, about what’s on our minds […] think about my next vacation.	Recovery
	DS10: The fact that you just sit there with your thoughts and reflect on things, that’s actually not so bad.	Recovery
	LS01: wait (sighs) for the end of the workday.	Recovery
	LS10: I leave work early.	Recovery
	DS03: Taking a longer lunch break or so […] I allow me to take a break.	Recovery
	DS04: It also happens that I sometimes drink a coffee here and there.	Recovery
	DS07: Sometimes I just listen to music and even dance a little bit to it […] I sit there and fall asleep for a while.	Recovery
	DS08: I enjoy having these downtimes [idle time]. That’s where I really enjoy my free time.	Recovery
	DS09: For example, I bring a book to read […] in my dressing room the ladies, uh, love to chat.	Recovery
	DS10: Idle time is also a bit of relaxation. I get myself a coffee or something to eat, and I visit a few other stands at the market.	Recovery
	DS03: I read a lot of newspapers, just online newspapers, or I text with my smartphone.	Cyberloafing
	DS04: I‘m using my smartphone from time to time.	Cyberloafing
	DS05: Well, you also look up private things on the Internet.	Cyberloafing
	DS07: Also a few private things, phone calls […] yeah, or I chat, answer messages.	Cyberloafing
	DS08: I just look at something on the computer, look on the Internet […] At the time we do not yet have a guilty conscience, we look also for vacations, for stocks, whatever.	Cyberloafing
	LS08: I actually, uh, read through online articles, mostly via Facebook,	Cyberloafing
	LS10: [Take] out the phone and see if there’s anything that needs to be done right now, or take care of private things for a moment.	Cyberloafing

### Objective and Subjective Idle Time

Interviewee 1 is a 29-year-old business consultant who sometimes experiences up to 7 h of idle time during an 8 to 9 h working day. His statements provide support for the aversive nature of subjective idle time:

*I really feel ashamed when my project manager is asking me about my progress, and I have to tell him that my hands are tied, and I am incapable of acting. You know, because I cannot, I cannot do anything about it. I think I am expected to work and to deliver and that’s why I feel ashamed.*

The second interviewee is a 37-year-old man, working in the automobile industry. His job is to maintain the machines and technical equipment in the factory. A computer system sends him a signal if there are any technical difficulties that need to be fixed. Sometimes they can be repaired immediately, but most of the time he has to wait until after the machine’s working hours, because he can only repair it when it is shut down.

Interviewee 3 is a 26-year-old customer service assistant. She already quit her old job because she experienced too much idle time. In her new position the experience of idle time is only slightly better. Her responsibility is to deal with customers who visit the company in person; sometimes she may support the service team that answers customer phone calls. She reported that, on a typical work day, she would get no visitors and three phone calls over a period of eight working hours.

Finally, Interviewee 4 is a self-employed restaurateur who described idle time as time that did not pass:

*The time is still there … even if there are no customers. I cannot serve anyone because no one is in the restaurant. But the time still is ticking.*

### Idle Time Antecedents

In the job of the business consultant, idle time can be caused by missing information, which is one often cited work constraint. Interviewee 1 cannot continue working because he is waiting for customer information or decisions. He said:

*I mean, [ … ] I have nothing to do. Why am I here in the first place and why do I get this huge paycheck? Just for being present and having nothing to do? I just sit here and wait for customer information.*

Asking him about the reasons for idle time he replied that it is an overestimation of personal and temporal resources. There are too many people in charge, and they have deadlines too far away. Moreover, it happens on a daily basis that he is only working on one project at a time, so if there is a constraint it is impossible for him to continue working.

For the car production machine maintenance engineer, idle time occurs due to machine working hours. Even if the computer system indicates a technical difficulty, most of the time Interviewee 2 cannot fix it right away.

Our last two example interviews resemble each other regarding idle time antecedents. For the customer service assistant and the restaurateur the cause of idle time is a lack of customers. Both interviewees cannot continue their work tasks because they have to wait for clients.

### Idle Time Consequences

Idle time consequences are best illustrated by direct quotes. Interviewee 1, the business consultant, revealed that this period of time is exhausting and provoking a high strain. He added:

*I am bored, insecure about being dismissed, unchallenged and I feel unseen. I would wish for appreciation of my work and capabilities. I think, I could do more, I feel not appreciated.*

Interviewee 2, the car production machine maintenance engineer already has turnover intentions:

*I think, I would like to have another job. I just feel … I feel tired. Imagine, you just feel exhausted and sit on a couch, that’s how I feel every day. For me, it’s not a dream job, nothing where I get up in the morning and feel enthusiastic or where I think ‘That’s what I wanted to do all along’. Nobody is really happy with this job at my company. It’s depressing.*

The customer service assistant, the third interviewee, talked about impending depression:

*I feel depressed. Sometimes, when I go home, I just start crying because I’m at a dead end. This makes me feel, I don’t know, not really sad, but unsatisfied in a way. When I only sit there and wait for the time to pass, I start to panic. It starts in the morning; it pains me to get in the car because I know [starts crying], because I know this day is going to be unbearable. I don’t know, it’s elusive how lousy it makes you feel, how it wears you out if you have nothing to do. I don’t want to get paid for doing nothing, for just sitting around on a chair and waiting for the time to pass so I can leave and go home.*

The last interviewee, the self-employed restaurateur mentioned fundamental existential fears:

*If there are no customers, I think about losses … not only financial losses. I’m talking about the fact that I work for nothing, and that I have to throw away food for example.*

### Proactive and Adaptive Strategies

We asked all interviewees about possible strategies to prevent or deal with idle time. The business consultant answered that he started to save up some tasks for these times, tasks which he could have accomplished some time earlier. Additionally, he talked about proactively asking co-workers if they needed help or doing something good for the organization, such as improving the website. In contrast, as adaptive strategies he mentioned taking longer breaks, drinking tea or coffee, running errands, as well as reading online newspapers, or leaving work early. As we asked him what he would change about his working environment, he replied that he would work on more than one project:

*The difficulty is the lack of control. The management decides who is working where and when. We only work for one customer at a time; I would love to have more overarching projects.*

Interviewee 2 also said that he tries to use proactive strategies and maintains lists of material or equipment. He tries to learn something about the functionality of the machines or chats with co-workers. When there is enough time left, they have longer chats not only about work, but about their private lives too. He said that from time to time he was really enjoying downtime periods. Because he is in a room with a computer, it may happen that he browses the internet, searching for travel destinations or stock prices.

The customer service assistant tries to work as slowly as possible by expanding the time needed to serve single customers. This is not easy for her because she does not want to waste her customers’ time. On some days she can support her colleagues by answering the phone and give customer support via phone or she tries to find something to do. She said she talked with her co- employees about work, cleaned her desk, and watered the plants, anything that helped not sitting around with nothing left to do. Most of the time, she replied, she was just sitting there and waiting for the time to pass. We then asked her why she would act in this specific way. She answered that she had no flexibility in her job, that she could not leave her workplace that she was asked to sit there and be present for potential customers.

The restaurateur said that he tried to cope with the negative feelings caused by idle time by turning the music on and getting himself in a positive mood. Sometimes he tries to dance the negativity away. Another thing for him to do during idle time is planning his next workdays and projects:

*I often think about my restaurant and how I can improve things. What can I change? What can I do better? How can I improve my work?*

We collected the qualitative interviews to provide preliminary support for the propositions of our conceptual model. We found initial evidence that idle time occurs in different industries and manifests itself very differently in duration and frequency. Overall, all of our propositions received some support by the data gathered in the interviews. In addition, the interviews provided us with numerous ideas for future research, which we will address in the discussion section, next.

## Discussion and Implications for Future Research

We introduced this paper with the question of whether 40 h of work a week may be “too much.” Indeed, survey results from the U.S. and Europe showed that employees do not always fill their entire working hours with work tasks (Brodsky & Amabile, 2018; Green & McIntosh, 2001). We assumed that, in many cases, somewhere in a “9 to 5” workday there must be phases of non-work. At the same time, most conceptual and empirical research on non-work periods at work has focused on breaks and interruptions (Baethge et al., 2014; Fritz et al., 2013). The important phenomenon of idle time, a period of involuntary downtime at work, has been largely neglected. Interestingly, twenty-five years elapsed between the first mention of a phenomenon similar to idle time, that is, boredom (Fisher, 1993), and the most recent investigations on idle time (Brodsky & Amabile, 2018; Lei et al., 2019). The latter indicate that idle time is a highly aversive experience, occurring in a wide range of occupations, that has significant consequences for both, individuals and organizations. Nevertheless, no scientific work has yet dealt in more theoretical depth with idle time. We have addressed this gap in the present paper and, thereby, contribute to the literature in important ways. First, we clarified the idle time definition, highlighting the duality between objective and subjective idle time. In doing so, we also distinguish idle time along several important dimensions from constructs that have been in the focus of prior research, including breaks, interruptions, procrastination, and withdrawal behavior. On the basis of action regulation theory, we then developed propositions that explain how idle time arises and which impact it can have on occupational well-being and task performance. We have further addressed possible strategies that employees may use for preventing or coping with idle time. Finally, we examined our propositions with a qualitative interview study demonstrating that idle time is a practically important phenomenon with potentially severe consequences.

We acknowledge that our conceptual model does not take into consideration all potentially existing predictors. For instance, we did not distinguish between objective and subjective idle time in the conceptual model. Since objective idle time is always a consequence of work constraints, the model focused on the path between work constraints and subjective idle time. The interviews, however, showed us that the distinction between subjective and objective idle time is conceptually useful. For example, in some interviews the experience of idle time was not immediately described, but became apparent over the course of the interview. One of the reasons for this was that people reported different behavioral strategies. Thus, in their perception, idle time did not occur because they were proactive throughout the idle time period. However, when asked whether there were situations with nothing to do at certain times, interviewees agreed.

With regard to behavioral strategies, it should be noted that they were represented in a rather simple way in our model. We roughly divided the strategies into proactive and adaptive strategies and gave two examples of each. There may be other strategies or responses to idle time that differ from these categories. For example, several interviewees mentioned that they sometimes leave work earlier, thereby shortening their working hours. Nevertheless, they would describe their work situation as idle because they were often confronted with having nothing to do. Furthermore, idle time may result in psychological reactance and, as a consequence, counterproductive work behavior. Considering the model of voluntary work behaviors, which describes organizational constraints as antecedents of counterproductive work behavior, it seems likely that idle time could play a role in the emergence of counterproductive work behavior as well (Miles et al., 2002; Spector & Fox, 2010).

With regard to the effects of idle time in the conceptual model, we specified occupational well-being and task performance as key outcomes based on their central role in action regulation theory (Zacher & Frese, 2018). Due to the aversive nature of idle time, individuals’ job satisfaction, life-domain balance, and turnover intentions might also be affected. Some interviewees indicated that they were thinking about changing jobs or already had done so. Another good example of this arises from the interview with the customer service assistant, where idle time even affected overall life satisfaction. Finally, the interviews not only provided useful suggestions regarding additional variables that could be added to the proposed categories within the model (e.g., the category of proactive strategies), but also that there may be other external conditions that might affect idle time, its occurrence, and how employees deal with it. Next, we offer several ideas in this regard.

### Theoretical Implications

Our conceptual model and propositions establish a basis for further investigations of idle time. In this section, we advance how situational, individual, and organizational factors might influence the proposed effects on and of subjective idle time. To this end, we focus on job autonomy and demands as potentially relevant situational working conditions; conscientiousness, cognitive appraisal, and proactive personality as key individual difference characteristics; and leadership, monitoring, and person-environment fit as organizational factors.

#### The Role of Job Autonomy and Job Demands

Job autonomy and job demands are two frequently investigated job characteristics (Bakker & Demerouti, 2017). Autonomy involves decisions over tasks, methods, and work schedules and allows for regulation possibilities (Frese & Zapf, 1994). If autonomy is high, employees have more opportunities to regulate their actions and use their skills and abilities (Zacher & Frese, 2018). On the one hand, autonomy helps to reduce the stressfulness of tasks-related stressors, such as work constraints (Fox et al., 2001). On the other hand, autonomy allows more opportunities to engage in proactive and adaptive strategies to prevent idle time or to cope with it. With high autonomy, individuals are enabled to choose adequate actions for situations, which helps them work more efficiently (Frese & Zapf, 1994). Consistently, higher levels of job autonomy were found to be associated with higher levels of proactive behaviors (e.g., Binnewies et al., 2009). In contrast, low levels of autonomy seem to be the most important hindering factor when it comes to proactive behavior at work (Parker et al., 2010). Focusing on adaptive strategies, evidence supports a relationship between autonomy and recovery, as well as cyberloafing (e.g., Mercado et al., 2017; Rodriguez-Muñoz et al., 2012). A meta-analysis showed a positive relation between autonomy and cyberloafing, suggesting that people who are empowered at work are more likely to cyberloaf (Mercado et al., 2017). This implies that individuals with higher job autonomy are more likely to either choose to prevent the experience of idle time or to cope with it. Thus, autonomy may have a positive effect on both, proactive and adaptive strategy use.

Job demands are categorized as those work aspects (physical, social, or organizational) that require effort and are linked to psychological costs (Demerouti et al., 2001). Some of them overlap conceptually, or are strongly linked with work constraints (Pindek & Spector, 2016). For example, a lack of equipment may hinder employees in fulfilling their work tasks, which in turn leads to a higher workload afterwards. Alternatively, work constraints can be seen in the context of hindrance and challenge work demands (Lepine et al., 2005). Hindrance demands, such as role conflict, impede goal achievement and overlap conceptually with work constraints. They are likely to trigger passive or coping behaviors (Lepine et al., 2005). Challenge demands, such as time pressure and high responsibility, encourage employees to overcome obstacles and prompt active problem-solving behaviors (Lepine et al., 2005; Sonnentag & Spychala, 2012). Moreover, action regulation theory suggests that challenging tasks (i.e., regulation requirements) contribute to personal development and learning at work (Zacher, 2017). Challenging tasks enable employees to experience all action regulation phases and levels (Frese & Zapf, 1994; Hackman & Oldham, 1976). It is likely that hindrance and challenge demands have an influence on employees’ prevention and/or coping strategy use during idle time at work.

Finally, action regulation theory shows that work tasks have to be challenging (i.e., regulation requirements) and provide control (i.e., regulation possibilities) to reduce strain, and to improve well-being and learning. Job demands-resources theory (Bakker & Demerouti, 2017) and conservation of resources theory (Hobfoll, 1989) support this relationship by proving job autonomy to be useful when demands are high. The combination of high demands and high autonomy motivates employees to engage in new behaviors and to learn new things (Karasek, 1979). It is likely that job autonomy, job demands, and their interaction influence not only the occurrence of work constraints, but also the way employees deal with idle time.

#### The Role of Individual Difference Characteristics

We focus here on conceptually relevant personality traits, including conscientiousness, negative affectivity, and proactive personality. Conscientiousness is one of the Big Five personality traits and encompasses ambitiousness and responsibility toward ethical standards and toward the consequences of one’s behavior (Costa & McCrae, 1992). Highly conscientious people are ambitious, diligent, and goal oriented (Hambrick & McCord, 2010). As the business consultant revealed in the interview, he felt ashamed of making no progress. Some other interviewees supported that feeling and added that idle time has an impact on them because they are highly conscientious and are willing to work. Employees who are highly conscientious might feel that idle time has a greater impact on occupational outcomes. A review on the influence of personality traits on occupational well-being revealed that conscientiousness is strongly related to work engagement (Mäkikangas et al., 2013). S. Grant and Langan-Fox (2007) found that conscientiousness moderates the relationship between role ambiguity (i.e., regulation uncertainty, Frese & Zapf, 1994) and job satisfaction such that the negative effect of role ambiguity on job satisfaction was diminished by conscientiousness. Moreover, conscientious persons do not want to waste time as it could be experienced during idle time, and might engage in proactive strategies (Bowling & Eschleman, 2010; Watson & Hubbard, 1996). Specifically, in a meta-analysis Colquitt et al. (2000) found conscientiousness to be strongly related to motivation to learn. For our conceptual model, we would expect conscientiousness to strengthen the relationship between subjective idle time and occupational well-being. Moreover, highly conscientious individuals should more likely engage in proactive strategies to prevent idle time in the first place.

Another personal characteristic that may influence the experience of well-being and coping in the context of idle time is negative affectivity. Negative affectivity is very similar to the construct of neuroticism or emotional stability as one of the Big Five personality traits (Costa & McCrae, 1992). People with a high negative affectivity are more likely to experience strain from stressful events, such as idle time (Watson & Clark, 1984). Cognitive appraisal is a concept explaining why some people experience situations in a more threatening way than others (Hambrick & McCord, 2010). Cognitive appraisal determines whether an event is experienced as stressful or not (Lazarus & Folkman, 1984). For some employees, idle time may have huge consequences on well-being and job satisfaction while for others this impact might be rather low. Some of the interviewees explained that they did not always feel stressed or exhausted during idle time, but that they could enjoy these periods from time to time. Moreover, re-appraising situations as not personally relevant may help coping with stressful events, such as idle time (Lazarus & Folkman, 1984).

Finally, we proposed in the conceptual model that proactive behavioral strategies would help to prevent the subjective experience of idle time. However, proactive personality could have an influence too. Proactive employees tend to identify opportunities and initiate active behaviors to make significant changes to their environment (Seibert et al., 1999). A meta-analytic review showed the significant relationships between proactive personality and different types of proactive behaviors (Fuller & Marler, 2009). Additionally, Bakker et al. (2012) found links between proactive personality and job crafting behavior. People with a proactive personality should therefore engage in proactive behavioral strategies to a greater extent than people with low proactive personality, and thus they should be more likely to prevent subjective idle time than their more passive counterparts.

Beyond these personality traits, other individual characteristics and differences in beliefs may play a role with regard to idle time. For instance, it might be of interest how demographic variables (e.g., age, gender, education), stress-relevant personality traits (e.g., impulsivity, self-control), and beliefs (e.g., core self-evaluations, psychological capital) impact the subjective experience of idle time and how individuals deal with it. First and with regard to the prevalence of idle time, our sample was heterogeneous in age and gender, as well as education. This is consistent with the samples in the studies of Brodsky and Amabile (2018) and Lei et al. (2019). Thus, we assume that individuals experience idle time regardless of age, gender, or education. We also did not find any demographic differences in the strategies used in our interviews. It is important to note, however, that it was not the focus of our interviews to examine demographics as potential moderators in the idle time processes. It might therefore be useful to conduct further studies that focus on demographic variables in connection with idle time. Second, stress-relevant personality traits might be relevant with regard to individuals’ responses to aversive events, such as idle time. It is conceivable that, for example, self-control plays a role in dealing with idle time. Self-control entails the ability to react to stressful events in a well-adjusted way (according to certain rules, morals, etc.; Baumeister et al., 2007). Third, core self-evaluations as one example for personal beliefs might influence the perception of and coping mechanisms with idle time. In a meta-analysis, Chang et al. (2012) report that core self-evaluations not only influence the perception of job characteristics (e.g., work constraints). They also found a negative relationship between core self-evaluations and harmful stressors that require coping (e.g., idle time). In summary, our conceptual model could be complemented by the above mentioned relevant constructs. Further theoretical and empirical work is needed to extend our conceptual model accordingly.

#### The Role of Organizational Factors

Regardless of situational and personal factors, factors in the organizational work environment, including leadership, monitoring, and person-environment fit, may influence idle time processes. Leadership may have direct effects on work constraints and work-related outcomes, as well as on strategy use. Regarding work constraints, leaders may impede goal achievement by not giving feedback or necessary information (Peters & O'Connor, 1980). Moreover, leaders are responsible for organizational rules and procedures working out the way they should. Regarding work-related outcomes, leadership was found to have meaningful effects on employees’ mental health (Montano et al., 2017). Especially, transformational leadership positively affected followers’ mental health. Transformational leaders motivate and inspire their followers, help them grow and exceed the expected (Bass, 1985), and empower their followers to be proactive (Steinmann et al., 2018). For proactive behavior at work, it is important to have a supportive environment and to not worry about risks or negative consequences of proactive behavior (Wu & Parker, 2017). Not only leadership styles, but also leaders as role models may influence employees’ strategy use. For example, Sonnentag and Schiffner (2019) found that leader psychological detachment from work is related to follower psychological detachment from work, which implies that leaders act as role models when it comes to recovery strategies at work. Regarding our conceptual model, leaders could influence the occurrence of idle time on the one hand. On the other hand, they could encourage employees’ investment in proactive behavioral strategies.

Another organizational context variable may be monitoring, as pointed out by Brodsky and Amabile (2018). They stated that individuals tend to stretch their work over the time available to not seem unproductive. Leaders’ evaluation of employees is not only based on the quality of work, but rather biased by the time employees are observed working (Chinander & Schweitzer, 2003; Elsbach et al., 2010). Even some of the respondents from our interviews said that they would act in another way when their manager was around. How monitoring affects strategy use during idle time could be explained by two mechanisms: monitoring reduces individual autonomy, so we assume that individuals would show both less proactive and less adaptive strategies. On the other hand, individuals always want to seem busy at all time, especially since this has an impact on supervisor evaluation of their performance. Thus, it is possible that individuals show more proactive behavioral strategies during idle time under monitoring.

Moreover, one of the interviewees explained that he is fine with being paid for sitting around and having nothing to do because the organizational values do not fit his own. The match between personal and organizational values is manifested in the person-environment fit (P-E fit; Edwards et al., 1998). P-E fit was found to positively influence not only job satisfaction and organizational commitment, but performance and career success as well (Kristof-Brown et al., 2005; Su et al., 2015). Evidence on the influence of P-E fit on work behaviors is rather weak. However, a better match between personal and organizational values should prompt more proactive behavior at work (Podsakoff et al., 2000; Yu & Davis, 2016). Thus, we assume that a high P-E fit increases individual proactive behavior strategies during idle time.

### Methodological Implications

Even though we found some preliminary support for our conceptual model in our qualitative interview study, it is necessary to examine the propositions using quantitative research designs. We suggest developing and validating a reliable and valid idle time scale. Brodsky and Amabile (2018) used single items to measure idle time in their investigation (i.e., frequency and duration of idle time at work). There are two difficulties with these items. First, participants have to gain a clear understanding of the definition of idle time. They must realize that idle time is a period that is involuntary, caused by external factors, and makes it impossible to continue working. Otherwise, it cannot be ensured that individuals report idle time or rather similar constructs, such as breaks or interruptions. Second, both items focus on objective but not on subjective idle time. Asking employees about objective idle time entails the possibility that participants who effectively used proactive strategies and dealt with idle time do not report experiencing it. A novel scale could include items on both objective and subjective idle time. Moreover, research could examine the differences of idle time and related constructs, such as breaks and interruptions, in a quantitative study.

We suggest examining the propositions of our conceptual model stepwise and with multiple methods. Brodsky and Amabile (2018) used an experimental design to investigate how idle time influences performance outcomes. In a replication of this study, researchers could focus not only on task performance, but also on well-being and work attitudes, such as job satisfaction. Additionally, the duration of idle time could be variated, and the subjective experience of idle time could be included as a mediator. To garner support for the propositions on strategy use, scholars could conduct an experimental vignette study. With a vignette design it is possible to specifically manipulate constraints and idle time, as well as working conditions to separate their effects on behavioral strategy use. Participants would imagine themselves in an idle time scenario and specify which strategies they would use.

To test the causal effects of idle time in a natural field setting, research could adopt longitudinal and diary study designs (Beal, 2015; Ployhart & Vandenberg, 2010). Event sampling methods could help to understand the immediate effects of work constraints and idle time on well-being outcomes, as well as the effects of strategy use on the subjective experience of idle time. Diary studies over a short period could indicate how changes in idle time influence changes in occupational well-being and task performance on a daily level. Additionally, this design would allow examining the influence of daily changing working conditions (i.e., job demands and autonomy) on idle time processes and strategy use. Furthermore, we suggest employing longitudinal studies over long periods, such as over several months or years to shed light on long-term outcomes, including turnover intentions or employee health.

Finally, to combine the advantages of both, experimental and field studies (i.e., high internal and external validity), scientist could carry out an intervention study with a waiting control group. It would be possible to examine the effectiveness of proactive and adaptive strategy use when experiencing constraints and idle time. Specifically, an intervention study could examine the impact of a change in employees’ strategy use before and during idle time on work outcomes. The training could involve theoretical input on idle time and strategy use, as well as practical exercises to train proactive and adaptive strategies.

## Conclusion

The overarching goal of this paper was to theoretically advance the notion of idle time. Based on action regulation theory, we developed a conceptual model on the antecedents and consequences of subjective idle time at work. We proposed that work constraints decrease occupational well-being and task performance, and that these direct links are mediated by the subjective experience of idle time. Moreover, the strength of these effects is expected to be influenced by employees’ use of proactive and adaptive strategies. We garnered initial support for our propositions from results of a qualitative interview study. We found that idle time is a phenomenon occurring in a wide range of occupations and significantly affecting individuals’ working experience. With this conceptual development paper, we laid the foundation for future theory-based investigations on idle time. Future research should on the one hand examine the suggested propositions empirically. On the other hand, scholars should focus on situational (i.e., job autonomy and demands), individual (i.e., conscientiousness, negative affectivity, and proactive personality), and organizational (i.e., leadership, monitoring, and P-E-fit) boundary conditions.

## Data Availability

The data and materials (interview manual, coding schemes, transcripts) of this study are openly available at the Open Science Framework: https://osf.io/9xm7t/.
